# Theoretical Analysis and Research on Support Reconstruction Control of Magnetic Bearing with Redundant Structure

**DOI:** 10.3390/s25144517

**Published:** 2025-07-21

**Authors:** Huaqiang Sun, Zhiqin Liang, Baixin Cheng

**Affiliations:** School of Mechanical and Automotive Engineering, Liaocheng University, Liaocheng 252059, China; 2022402748@stu.lcu.edu.cn (H.S.); 2021400357@stu.lcu.edu.cn (Z.L.)

**Keywords:** magnetic bearing, redundant structure, fault-tolerant control, electromagnetic force

## Abstract

At present, the redundant structures are one of the most effective methods for solving magnetic levitation bearing coil failure. Coil failure causes residual effective magnetic poles to form different support structures and even asymmetrical structures. For the magnetic bearing with redundant structures, how to construct the electromagnetic force (EMF) that occurs under different support structures to achieve support reconstruction is the key to realizing fault tolerance control. To reveal the support reconstruction mechanism of magnetic bearing with a redundant structure, firstly, this paper takes a single-degree-of-freedom magnetic suspension body as an example to conduct a linearization theory analysis of the offset current, clarifying the concept of the current distribution matrix (CDM) and its function; then, the nonlinear EMF mode of magnetic bearing with an eight-pole is constructed, and it is linearized by using the theory of bias current linearization. Furthermore, the conditions of no coils fail, the 8th coil fails, and the 6–8th coils fail are considered, and, with the maximum principle function of EMF, the corresponding current matrices are obtained. Meanwhile, based on the CDM, the corresponding magnetic flux densities were calculated, proving that EMF reconstruction can be achieved under the three support structures. Finally, with the CDM and position control law, a fault-tolerant control system was constructed, and the simulation of the magnetic bearing with a redundant structure was carried out. The simulation results reveal the mechanism of support reconstruction with three aspects of rotor displacement, the value and direction of currents that occur in each coil. The simulation results show that, in the 8-pole magnetic bearing, this study can achieve support reconstruction in the case of faults in up to two coils. Under the three working conditions of wireless no coil failure, the 8th coil fails and the 6–8th coils fail, the current distribution strategy was adjusted through the CDM. The instantaneous displacement disturbance during the support reconstruction process was less than 0.28 μm, and the EMF after reconstruction was basically consistent with the expected value.

## 1. Introduction

In recent years, magnetic bearings have been increasingly used in support units of rotating machines, such as magnetic levitation blowers, magnetic levitation molecular pumps and control moment gyroscopes, because of their advantages, which include low vibration, no mechanical friction and adjustable support stiffness [1,2,3,4]. SKF uses magnetic motors in ventilation blowers, reducing noise levels by 30% and energy consumption by 40%. The magnetic levitation centrifugal compressor of S2M Company is applied in natural gas transportation, with a maximum power of up to 30 MW. The GE Company of the United States has applied magnetic bearings in integrated compressors, with a maximum output power of 15 MW and a maximum rotational speed of 11,000 RPM. Contactless support eliminates the need for lubrication and maintenance, significantly reducing friction losses [5]. Through a differential control strategy, the symmetrically constrained stator structure in conventional magnetic bearings generates counter-rotating magnetic fields to levitate the rotor, maintaining its equilibrium via combined magnetic forces [6].

Currently, the research work of magnetic bearings mainly focuses on imbalance vibrations and harmonic suppression. Due to unavoidable issues such as rotor manufacturing defects, magnet runout and sensor jitter, significant rotor harmonic oscillations can easily occur, causing excessive harmonic currents in the control system and jeopardizing the stability of the magnetic bearing system. In addition, because the inertia axis of the rotor does not completely coincide with the geometric axis, the magnetic flux distribution in the air gap between the rotor and stator becomes uneven, leading to unbalanced magnetic pull. In response to the above issues, scholars have conducted relevant research on the suppression of periodic disturbances in rotors. H. Zhang et al. [7] analyzed the formation mechanism of harmonic vibration in the magnetic bearing system, designed a nonlinear adaptive control algorithm, obtained the separation signals of rotor imbalance and sensor jitter, and effectively suppressed the harmonic vibration of the rotor by compensating the displacement stiffness at different rotational speeds. To address the challenge of sensor jitter, Y. Chen et al. [8] proposed a displacement sensor self-calibration method for magnetic bearing current control. Experiments conducted on a magnetic levitation CMG platform have verified the method’s ability to compensate for sensor sensitivity and zero-point drift. Moreover, the stability of high-speed magnetic bearings is critically influenced by the gyroscopic torque that is induced by base motion. By analyzing the formation mechanism of the gyroscopic effect in the magnetic bearing-rotor system, C. Wang et al. [9] developed a linear extended state observer with an adaptive notch filter. Through dynamic disturbance prediction and gain compensation, the interference of gyroscopic torque in high-speed magnetic bearings can be effectively suppressed. Due to the temperature rise induced by the high-speed rotation of the magnetic bearing, the coil resistance increases, and the supporting stiffness is weakened; thus, low-frequency vibration is triggered. W. Ma et al. [10] used an online identification algorithm based on the forgetting factor recursive least squares method to obtain the resistance of the magnetic bearing coil and perform real-time stiffness compensation, effectively suppressing the low-frequency fluctuations of the rotor. To reduce the complexity of the magnetic bearing system, H. Xu et al. [11] proposed a sensorless control method for imbalance vibration. The utilization of a vibration suppression compensator, in conjunction with a phase-locked loop-based rotor frequency estimator, enabled the tracking and estimation of the rotor frequency. This approach effectively suppressed rotor displacement vibrations. In addition, P. Zhang et al. [12] conducted research on rotor vibration issues under base excitation conditions, proposing a base acceleration feedforward compensation algorithm. By combining the rotor dynamic model, researchers generated optimal compensation currents to reduce rotor vibration. Experimental results showed that the maximum vibration reduction rate of this scheme reached 85%. Han Xue [13] analyzed the electromagnetic pulling force caused by the dynamic eccentricity of the rotor, designed an adaptive peak filter to suppress the high-frequency noise experienced by the rotor and formed a cascade structure with the state observer to accurately estimate the magnetic pulling force disturbance. Predictive compensation was carried out throughout the full rotational speed range. Simulation results show that this method significantly reduces the rotor vibration caused by the magnetic pulling force.

Due to the inherent nonlinearity of the magnetic bearing system and the system uncertainty caused by external disturbances, Xu Yunlang et al. [14] proposed an adaptive sliding film control method (CASMC) to improve the accuracy of levitation control. This method includes an equivalent controller, a composite neural network compensator and an adaptive switch controller. Experimental results show that the CASMC method has better dynamic response and stronger anti-interference ability. However, neural network compensators require complex computational models, resulting in increased system latency. Due to the inertial coupling and gyroscopic effect of magnetic bearings in the four degrees of freedom in the radial direction, it seriously affects the control accuracy and stability. To address this issue, Chai ChangPeng et al. [15] proposed a decoupled controller of the generalized extended state observer (GESO), uniformly modeling the inertial coupling, gyroscopic effect, and external disturbances as the total disturbance. Dynamic compensation is achieved by using GESO. The simulation results show that this method can effectively improve the control accuracy and system stability under step, sine and disturbance pulses.

Repetitive control is also an effective method for eliminating harmonic vibrations. The objective of conventional repetitive control algorithms is to eliminate all periodic errors. However, in nonlinear load systems such as magnetic bearings, odd-order harmonic components are dominant. To this end, Peiling Cui et al. [16,17,18] developed a dynamic model of the magnetic bearing system, designed a dedicated repetitive controller for it, and performed phase compensation through a filter to ensure the system’s stability across a specified frequency range. Additionally, they devised a second-order repetitive controller and performed parameter optimization to enhance the system’s convergence performance. They achieved multi-frequency vibration suppression of the magnetic bearing system, thereby improving the support accuracy of the rotor. Moreover, magnetic bearing systems are susceptible to uncertain disturbances under complex operating conditions. To address this, the authors of [19] proposed a feedback linearization-extended state observer into PID control. By transforming the nonlinear system into a linear integral chain, the strategy enables the real-time estimation of total disturbances and implements compensation, effectively suppressing rotor wideband disturbances and enhancing the rotor’s anti-interference capability.

However, the above-mentioned research on the harmonic vibration suppression of magnetic bearings has primarily focused on magnetic bearings with symmetrical constraints. In such magnetic bearing systems, while some functional components malfunction, such as the coil fail or power amplifiers, the symmetrical constraints of them are disrupted. The function of coils where the components fail is lost, unable to provide the EMF required by the system [20,21,22]. To improve the stability of the magnetic bearing system and prevent the system from being affected by the disruption of symmetry constraints. Maslen and Meeker [23] proposed a magnetic bearing with a redundant structure to address the coil failure. In this structure, each magnetic coil is wound separately, which makes more efficient use of the bearing’s structural redundancy. EMF modeling and CDM solution are the key links to achieve the fault-tolerant control of a magnetic bearing with a redundant structure.

For EMF, Eric Maslen et al. [23] proposed the generalized bias current linearization theory, which solved the traditional stator dual problem of magnetic bearings. Through numerical optimization, they determined the optimal linearization scheme and current distribution strategy. To enhance the accuracy of the EMF model, Na and Palazzolo [24] designed an eight-pole heteropolar magnetic bearing and incorporated material reluctance into the magnetic circuit equations. Leveraging the Lagrange multiplier method, they optimized the EMF model and current distribution strategy, achieving fault-tolerant control under scenarios involving multiple coil failures. Considering the rotational loss of the heteropolar magnetic bearing laminated rotor and the hysteresis influence of the rotor material, Meekr et al. [25] used the eddy current model to predict the hysteresis loss of the magnetic bearing laminated rotor, which further improved the accuracy of the EMF model. The above research focuses on the control strategy of a single fault mode. To solve the multiple functional component failures, Noh et al. [26] designed a dynamic switching mechanism between actuator redundancy and sensor redundancy to achieve the synchronous fault-tolerant control of sensor failure and actuator failure and experimentally verified it on the turbomolecular vacuum pump. Cheng Xin et al. [27,28] conducted relevant research on the fault-tolerant control of weakly coupled magnetic bearings and proposed a dynamic magnetic flux compensation method based on adjacent magnetic poles. By reconstructing the local magnetic field distribution of the faulty magnetic poles, they achieved the reconstruction of the support characteristics after the failure. To simplify the bias current linearization solution procedure, Meeker and Maslen [29] proposed a method for analytical calculation using a small number of parameter indices. In the case of actuator failure, linearization of the EMF model can still be achieved, but it is only applicable to bearing states with an even number of uniformly spaced poles of equal area.

The CDM is the core of a magnetic bearing system to achieve multi-coil collaborative control. Its purpose is to dynamically adjust the current distribution strategy of each coil to meet the EMF required for rotor suspension. Regarding the EMF dynamic compensation problem of the magnetic bearings with an 8n-pole symmetrical structure under magnetic pole failure, Na and Palazzolo [30] proposed a CDM optimization method based on the Lagrange multiplier method. By introducing the EMF balance equation under constraint conditions, the global magnetic flux reconstruction under concurrent failures of five magnetic poles was theoretically achieved. A current control strategy for magnetic bearings based on the CDM and a displacement-current stiffness model for magnetic bearings with a redundant structure has been established. To reduce the controller power, Na and Palazzolo [31] optimized the CDM using the controller power constraints and the EMF linearization conditions and designed a fault-tolerant control strategy for current groupings for magnetic bearings. Based on the characteristic that the magnetic flux remains unchanged before and after the actuator fails, Na [32] used the Lagrange algorithm to handle the CDM solution problem and designed the corresponding current distribution controller. Cheng Xin et al. [33] took the current saturation as the limiting condition, hoped to obtain the minimum control current when the maximum EMF output was achieved and derived the optimal solution of the bias current coefficient in the control theory of magnetic bearings with a redundant structure. Furthermore, to solve the problem of excessive energy consumption caused by the current variation in multi-pole coils, S. Deng et al. [34] designed a multi-objective optimization control strategy and conducted simulation verification. X. Cheng [35] completed the software and hardware schemes of the controller and driver in accordance with the requirements of the fault-tolerant control of magnetic bearings with a redundant structure.

Based on the above analysis, at present, the research work on magnetic bearings without redundant structures mainly focuses on aspects such as rotor unbalanced vibration and harmonic current suppression. For magnetic bearings with redundant structures, previous studies have mostly focused on single-point faults or faults with symmetrical constraints. There is still no research on the asymmetric multi-point fault problem. Therefore, it is necessary to analyze the asymmetric fault support reconstruction mechanism of magnetic bearings with a redundant structure.

We assume that the CDM can achieve EMF reconstruction under the asymmetric support structure caused by coil failure and realize the stable suspension of the rotor through dynamic fault-tolerant control. This study answers the three key issues: (1) How to avoid coil failure through CDM implementation under the condition of the reconstruction of electromagnetic force? (2) How does fault-tolerant control maintain rotor stability during the support reconstruction process? (3) Why is redundant structure the solution for high-reliability magnetic bearings?

## 2. Modeling of EMF

### 2.1. Nonlinear EMF

**Figure 1:**
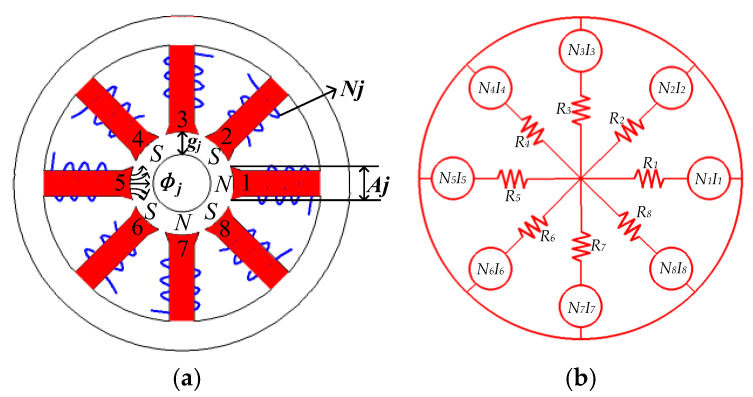
Eight-pole bearing arrangement (**a**) and equivalent magnetic circuit (**b**).

In this paper, an eight-pole bearing was designed with a redundant structural scheme of winding mode, as described in Figure 1a. Figure 1b shows the expression of its equivalent magnetic circuit.

In the current system, a single magnetic pole forms a magnetic field and couples with the magnetic fields of the other poles, which in turn creates an EMF. With the stator reluctance taken into account, the magnetic circuit equation is established from Ampere’s theorem:

(1) $$R_{j}\phi_{j}-R_{j+1}\phi_{j+1}=N_{j}I_{j}-N_{j+1}I_{j+1},j=1,2,\cdots ,n-1$$

(2) $$R_{j}=\frac{g_{j}}{\mu_{0}A_{j}}$$

where some parameters are defined as in Table 1.

The $g_{j}$ can be expressed as

(3) $$g_{j}=g_{0}-x\cos\theta_{j}-y\sin\theta_{j}$$

According to the law of conservation of magnetic flux, it can be obtained:

$$\sum_{j=1}^{n}\phi_{j}=0$$

where

(4) RΦ=NI

where $R=\left[\begin{array}{ccccc} R_{1} & -R_{2} & 0 & \cdots & 0\\ 0 & R_{2} & -R_{3} & \ddots & \vdots \\ \vdots & \ddots & \ddots & \ddots & 0\\ 0 & \cdots & 0 & R_{n-1} & -R_{n}\\ 1 & 1 & \cdots & 1 & 1 \end{array}\right]$, $\phi =\left[\begin{array}{c} \phi_{1}\\ \phi_{2}\\ \vdots \\ \phi_{n-1}\\ \phi_{n} \end{array}\right]$

$N=\left[\begin{array}{ccccc} N_{1} & -N_{2} & 0 & \cdots & 0\\ 0 & N_{2} & -N_{3} & \ddots & \vdots \\ \vdots & \ddots & \ddots & \ddots & 0\\ 0 & \cdots & 0 & N_{n-1} & -N_{n}\\ 0 & 0 & \cdots & 0 & 0 \end{array}\right]$, $I=\left[\begin{array}{c} I_{1}\\ I_{2}\\ \vdots \\ I_{n-1}\\ I_{n} \end{array}\right]$.

Assuming that the magnetic flux density is uniform in the gap, the flux matrix can be described as

(5) Φ=AB

Then

(6) $$B=A^{-1}R^{-1}NI=VI$$

where ***A*** is the diagonal matrix of the pole area, and ***B*** is the magnetic flux density matrix of the air gap.

Where ***V*** = ***A***^−1^***R***^−1^***N*** is defined as the flux density and current relation matrix, ***A*** denotes the magnetic pole area matrix.

Based on the above theory, the mathematical model of the EMF in the *x*, *y* direction of the magnetic levitation bearing with redundant structure can be obtained as

(7) $$\left\{\begin{array}{l} F_{x}(x,y)=-B^{T}\frac{\partial D(x,y)}{\partial x}B\\ F_{y}(x,y)=-B^{T}\frac{\partial D(x,y)}{\partial y}B \end{array}\right.$$

where D(x,y) represents the relationship between the magnetic pole area of the magnetic bearing and the air gap between the magnetic pole and the rotor, which is given as

(8) $$D(x,y)=diag\left[\frac{A_{j}g_{j}(x,y)}{2\mu_{0}}\right]$$

Finally, simultaneous Equations (2)–(8)~(2)–(11), the model expression for the nonlinear EMF generated by the magnetic levitation bearing in the *x* and *y* directions, respectively, can be obtained as follows:

(9) $$\left\{\begin{array}{l} F_{x}(x,y)=-I^{T}V^{T}D_{x}VI=I^{T}M_{x}I\\ F_{y}(x,y)=-I^{T}V^{T}D_{y}VI=I^{T}M_{y}I\\ D_{x}=\frac{A}{2\mu_{0}}\cdot diag[\begin{array}{cccc} \cos\theta_{1} & \cos\theta_{2} & \cdots & \cos\theta_{n} \end{array}]\\ D_{y}=\frac{A}{2\mu_{0}}\cdot diag[\begin{array}{cccc} \sin\theta_{1} & \sin\theta_{2} & \cdots & \sin\theta_{n} \end{array}] \end{array}\right.$$

$M_{x}$ and $M_{y}$ are defined as the relationship matrix between current and EMF.

(10) $$\left\{\begin{array}{c} M_{x}(x,y)=-V^{T}D_{x}V\\ M_{y}(x,y)=-V^{T}D_{y}V \end{array}\right.$$

From the analysis of Equations (9) and (10), the magnitude of the EMF acting on the rotor is not only related to the coil current and the air gap but also depends on the angle of the magnetic poles with respect to the *x*, *y* axis. This condition is not subject to the constraints of pole symmetry in radial maglev bearings, which provides a wider range of applications for fault-tolerant control of maglev bearings with redundant structures. Besides that, it can be found that the EMF, coil current and air gap are quadratic nonlinear relations. Supported by redundant magnetic levitation bearings, it faces a reconfiguration process of rotor levitation; the nonlinear nature of the EMF will inevitably cause instability of the rotor. Therefore, linearized modeling of nonlinear EMF with respect to rotor displacement and coil current is required, which in turn leads to the design of linear controllers that satisfy the magnetic levitation rotor near the balanced position.

### 2.2. Generalized Linearization of the EMF

**Figure 2:**
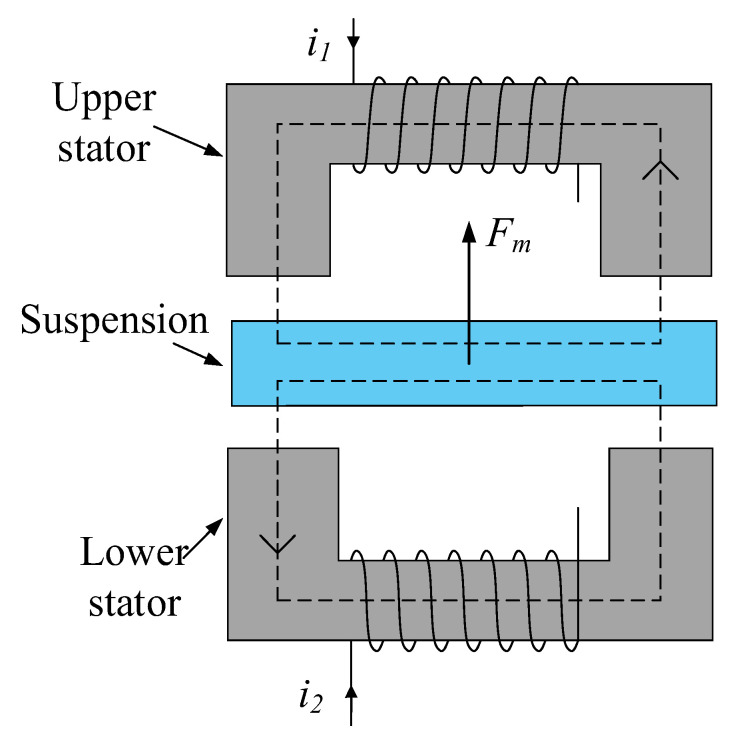
Single-degree-of-freedom magnetic levitation structure.

Bias current linearization is a common method to achieve linearization between EMF and currents. In order to control the suspension at a single degree of freedom, most magnetic levitation systems use pairs of relative electromagnet stators, as shown in Figure 2.

In Figure 2, the coils of the upper and lower stators were, respectively, fed with current. A magnetic circuit was formed between the stator and the suspended body to generate an EMF. The suspended body was suspended under the action of EMF. For the Maglev structure shown in Figure 2, the combined force on the suspended body can be expressed as

(11) $$F_{m}=c(i_{1}^{2}-i_{2}^{2})$$

where *c* denotes a constant related to the physical properties of the magnetic levitation structure. $i_{1}$ and $i_{2}$ represent the magnitude of current in the upper and lower stator coils, respectively.

In order to linearize the relationship between EMF and coil current, bias current coefficient and control logic current were introduced, which are defined as follows:

(12) $$\left\{\begin{array}{l} i_{1}=\left(C_{0}+i_{c}\right)/\left(2\sqrt{c}\right)\\ i_{2}=\left(C_{0}-i_{c}\right)/\left(2\sqrt{c}\right) \end{array}\right.$$

By combining Equations (11) and (12), we can obtain the following:

(13) $$F_{m}=C_{0}i_{c}$$

By analyzing Equations (12) and (13), we can see that, when the bias current coefficient *C*_0_ is a constant, the EMF *F_m_* generated by the levitated body is linearly related to the control current *i_c_*. The adjustment of *C*_0_ through the control system can generate EMF, and, at this point, the current in the coils of the upper and lower stators and EMF need to meet the following conditions:

(14) $$\left[\begin{array}{c} i_{1}\\ i_{2} \end{array}\right]=\frac{1}{2\sqrt{c}}\left[\begin{array}{cc} 1 & 1\\ 1 & -1 \end{array}\right]\left[\begin{array}{c} C_{0}\\ F_{m}/C_{0} \end{array}\right]$$

For the magnetic suspension structure shown in Figure 2, Equation (14) describes the relationship between the current of the two coils and the EMF. Here define $I={\left[\begin{array}{cc} i_{1} & i_{2} \end{array}\right]}^{T}$, $I_{c}={\left[\begin{array}{cc} C_{0} & i_{c} \end{array}\right]}^{T}$, and then Equation (14) is transformed into the following:

(15) $$I=\frac{1}{2\sqrt{c}}\left[\begin{array}{cc} 1 & 1\\ 1 & -1 \end{array}\right]I_{c}=WI_{c}$$

The symbol ***W*** in Equation (15) is considered to be the CDM between the currents in the two magnetic pole coils and the EMF, which reflects the mapping relationship between the actual current in the coils and the EMF. According to the above theoretical analysis, it is theoretically possible to linearize the EMF and current by using bias current linearization. A set of coil currents can be solved based on the CDM ***W*** and the determined EMF, and this mathematical relationship provides a theoretical guide for the controller design of the magnetic levitation system.

This is similar to magnetic bearings with redundant structures, although different support structure configurations have been formed due to partial magnetic pole failure; however, for each type of EMF generated by the support structure, its nonlinear EMF can be linearized using the bias current linearization theory [20].

By introducing bias current coefficient *C*_0_, control current vector $I_{c}$, and CDM ***W***, the EMF and current can be linearized. The EMF of a single magnetic bearing generally needs to be decomposed into components in the *x* and *y* directions; therefore, the control current vector $I_{c}$ is defined as

(16) $$I_{c}={\left[\begin{array}{ccc} C_{0} & i_{x} & i_{y} \end{array}\right]}^{T}={\left[\begin{array}{ccc} C_{0} & \frac{F_{x}}{C_{0}} & \frac{F_{y}}{C_{0}} \end{array}\right]}^{T}$$

where $i_{x}$ is the control logic current in the *x*-direction, and $i_{y}$ is the control logic current in the *y*-direction. $F_{x}$ and $F_{y}$ are the EMF on the rotor in the *x* and *y* directions, respectively. The CDM is defined as follows:

(17) $$W=[W_{b},\;W_{x},W_{y}]$$

where

(18) $$\left\{\begin{array}{l} W_{b}={\left[\begin{array}{cccc} a_{11} & a_{21} & \cdots & a_{n1} \end{array}\right]}^{T}\\ W_{x}={\left[\begin{array}{cccc} a_{12} & a_{22} & \cdots & a_{n2} \end{array}\right]}^{T}\\ W_{y}={\left[\begin{array}{cccc} a_{13} & a_{23} & \cdots & a_{n3} \end{array}\right]}^{T} \end{array}\right.$$

The current in each magnetic pole coil and the control current both meet the following relationship:

(19) $$I=W\left[\begin{array}{c} C_{0}\\ i_{x}\\ i_{y} \end{array}\right]=W_{b}C_{0}+W_{x}i_{x}+W_{y}i_{y}$$

For simultaneous Equations (9) and (19), the linearized expression for the EMF is as follows:

(20) $$\left\{\begin{array}{l} F_{x}(x,y)=I_{c}^{T}W^{T}M_{x}WI_{c}\\ F_{y}(x,y)=I_{c}^{T}W^{T}M_{y}WI_{c} \end{array}\right.$$

In the EMF, if the following relationship is satisfied,

(21) $$\left\{\begin{array}{c} W^{T}(x,y)M_{x}W(x,y)-Q_{x}=0\\ W^{T}(x,y)M_{y}W(x,y)-Q_{y}=0 \end{array}\right.$$

Among them,

(22) $$Q_{x}=\left[\begin{array}{ccc} 0 & 0.5 & 0\\ 0.5 & 0 & 0\\ 0 & 0 & 0 \end{array}\right],\;Q_{y}=\left[\begin{array}{ccc} 0 & 0 & 0.5\\ 0 & 0 & 0\\ 0.5 & 0 & 0 \end{array}\right]$$

Then the linearization between the EMF and the current can be achieved:

(23) $$\left\{\begin{array}{c} F_{x}=C_{0}i_{x}\\ F_{y}=C_{0}i_{y} \end{array}\right.$$

From the above analysis, for magnetic bearing with redundant structures, it can be seen that the linearization of the corresponding nonlinear EMF and control logic currents can be achieved simply by using a CDM that matches the support structure.

Comparing Equation (23) with Equation (9), it can be found that the bias current linearization method transforms the nonlinear CDM model expressed by multiple coil currents, and the air gap between the stator and rotor develops into a linearized form of the EMF and the control logic current.

### 2.3. Algorithm for Solving the CDM

Equation (21) can be equivalent to 12 constraint equations:

(24) $$\left\{\begin{array}{l} G_{1}(W)=W_{b}^{T}M_{x}W_{b}=0\\ G_{2}(W)=W_{b}^{T}M_{x}W_{x}-0.5=0\\ G_{3}(W)=W_{b}^{T}M_{x}W_{y}=0\\ G_{4}(W)=W_{x}^{T}M_{x}W_{x}=0\\ G_{5}(W)=W_{x}^{T}M_{x}W_{y}=0\\ G_{6}(W)=W_{y}^{T}M_{x}W_{y}=0\\ G_{7}(W)=W_{b}^{T}M_{y}W_{b}=0\\ G_{8}(W)=W_{b}^{T}M_{y}W_{x}=0\\ G_{9}(W)=W_{b}^{T}M_{y}W_{y}-0.5=0\\ G_{10}(W)=W_{x}^{T}M_{y}W_{x}=0\\ G_{11}(W)=W_{x}^{T}M_{y}W_{y}=0\\ G_{12}(W)=W_{y}^{T}M_{y}W_{y}=0 \end{array}\right.$$

This satisfies linearization and current saturation as constraints to maximize the minimum EMF at any angle of the output of the magnetic bearing. Assuming that the angle of the desired EMF F with the *x*-axis is β, for an n-pole magnetic bearing with redundant structure, the expression between the coil current of each pole and the EMF is given by

(25) $$I_{i}=a_{i1}C_{0}+\frac{F}{C_{0}}\cdot \left(a_{i2}\cos\beta +a_{i3}\sin\beta \right)\;\;\;\;0<i\leq n$$

Then the expected EMF is as follows:

(26) $$F=\frac{C_{0}(I_{i}-a_{i1}C_{0})}{a_{i2}\cos\beta +a_{i3}\sin\beta }\;0\;<\;i\;\leq \;n$$

From Equation (25), under the condition of coil current saturation constraint, the maximum value of the EMF depends on the CDM and the EMF angle when the bias current coefficient *C*_0_ is determined. If the EMG is maximized and the desired EMF is generated at any angle, only the angle corresponding to the minimum EMF needs to be solved; then, the output EMF at that angle is made to be maximized, and the corresponding CDM is obtained.

Set the current saturation value to Imax and construct the EMF objective function:

(27) $$\max F(W)=\underset{W_{0}\to W}{\max}\left[\underset{i=1,2,...,n}{\min}\frac{C_{0}(I_{\max}-a_{i1}C_{0})}{a_{i2}\cos\beta +a_{i3}\sin\beta }\right]$$

Through the above analysis, the theory of obtaining the minimum value of the objective function by using nonlinear equations and inequality constraints can be utilized to calculate the CDM. Organize Equations (26) and (27) into Equation (28).

(28) $$\left\{\begin{array}{l} \min\left[f\left(W\right)=\frac{1}{F_{ex}(W)}\right]\\ -\left|I_{\max}\right|\leq a_{11}C_{0},a_{21}C_{0},\cdots ,a_{n1}C_{0}\leq \left|I_{\max}\right|\\ F_{ex}(W)\geq 0\\ G\left(W\right)=0 \end{array}\right.$$

Using the fmincon function provided by Matlab, the CDM satisfying the above conditions was solved iteratively by giving the initial values. In the actual solving process, the iterations: 20, stepsize: 0.0036, algorithm: ‘interior-point’, the relative maximum constraint violation, 8.9 × 10^−4^ and Constraint Tolerance = 1.0 × 10^−6^. Then the linearization conditions were verified on the derived results until the constraints are satisfied. The CDM ***W***(0,0) of the rotor at the equilibrium position was found using numerical calculations.

## 3. Simulation Verification

### 3.1. The CDM Calculation

For the magnetic bearing and rotor that are studied in this paper, the structural parameters are shown in Table 2.

In order to verify the validity of the theory of support reconfiguration for magnetic bearing with redundant structures, this paper considers the following cases: no coil fails, the 8th coil fails, and the 6th and 8th coils fail. The corresponding current distribution matrices under the three working conditions are shown in Equations (29)–(31).

(29) $$W\left(0,0\right)=\frac{g_{0}}{4N\sqrt{\mu_{0}A}}\left[\begin{array}{ccc} 2 & 2 & 0\\ -2 & -\sqrt{2} & -\sqrt{2}\\ 2 & 0 & 2\\ -2 & \sqrt{2} & -\sqrt{2}\\ 2 & -2 & 0\\ -2 & \sqrt{2} & \sqrt{2}\\ 2 & 0 & -2\\ -2 & -\sqrt{2} & \sqrt{2} \end{array}\right]$$

(30) $$W{\left(0,0\right)}_{8}=\frac{g_{0}}{4N\sqrt{\mu_{0}A}}\left[\begin{array}{ccc} 4 & 2+\sqrt{2} & -\sqrt{2}\\ 0 & 0 & -2\sqrt{2}\\ 4 & \sqrt{2} & 2-\sqrt{2}\\ 0 & 2\sqrt{2} & -2\sqrt{2}\\ 4 & -2+\sqrt{2} & -\sqrt{2}\\ 0 & 2\sqrt{2} & 0\\ 4 & \sqrt{2} & -2-\sqrt{2}\\ 0 & 0 & 0 \end{array}\right]$$

(31) $$W{\left(0,0\right)}_{68}=\left[\begin{array}{ccc} -0.3705 & -0.8676\; & -0.1521\\ 0.1702\; & -0.0034 & -0.2830\\ 0.4855 & -0.0060 & 0.7401\\ 0.0905 & -0.0194 & -0.2830\\ -0.3795 & 0.8559 & -0.1335\\ 0 & 0 & 0\\ 0.4902 & -0.0052 & -1.0239\\ 0 & 0 & 0 \end{array}\right]$$

Based on the CDM, the EMF reconstruction can be calculated according to the current redistribution results of each coil under three working conditions.

### 3.2. The EMF Reconfiguration Validation

Set the desired EMF of the rotor at the balance position as *F_x_* = 8 N, *F_y_* = 0 N, bias current coefficient *C*_0_ = 4. According to the structural parameters of the magnetic bearing shown in Table 2, by means of the current distribution matrices Equations (29)–(31), the current values $I={\left[\begin{array}{cccc} I_{1} & I_{2} & \cdots & I_{8} \end{array}\right]}^{T}$ required by each magnetic pole coil in three working conditions (no coil fails, the 8th coil fails and the 6–8th coils fail) are obtained, respectively, and the calculation results are shown in Table 3.

From Table 3, it can be seen that the corresponding coil current values under the three working conditions are redistributed. In particular, the change in current that occurs in the 6th and 8th coils reflects the fault state of the coil. Under three working conditions, we analyzed the RMS current and power consumption of the magnetic bearing system. The system RMS current is shown in Table 4.

Based on the structural parameters of the magnetic bearing in Table 2, the coil resistance R was estimated to be 0.5 Ω. The power consumption of the magnetic bearing system is shown in Table 5.

From Table 4 and Table 5, it can be seen that, while the 8th coil fails, the current and power consumption increase sharply due to current distribution, which is used to compensate for the carrying capacity of the failed coil. While the 6–8th coils fail, the system’s power consumption decreases due to the failure of multiple coils.

The EMF of the analytic magnetic bearing with a redundant structure is generated by the magnetic field coupling of all magnetic poles. In order to calculate the actual EMF generated by a magnetic bearing with a redundant structure, according to Equation (6) and the structural parameters of magnetic bearings, the magnetic field strength Equation (32) can be obtained.

(32) $$B=\left[\begin{array}{l} \;\;\;\;\;\;0.1539\;-0.0220\;-0.0220\;-0.0220\;-0.0220\;-0.0220\;-0.0220\;-0.0220\\ \;-0.0220\;\;\;\;\;\;0.1539\;-0.0220\;-0.0220\;-0.0220\;-0.0220\;-0.0220\;-0.0220\\ \;-0.0220\;-0.0220\;\;\;\;\;\;0.1539\;-0.0220\;-0.0220\;-0.0220\;-0.0220\;-0.0220\\ \;-0.0220\;-0.0220\;-0.0220\;\;\;\;\;\;0.1539\;-0.0220\;-0.0220\;-0.0220\;-0.0220\\ \;-0.0220\;-0.0220\;-0.0220\;-0.0220\;\;\;\;\;\;0.1539\;-0.0220\;-0.0220\;-0.0220\\ \;-0.0220\;-0.0220\;-0.0220\;-0.0220\;-0.0220\;\;\;\;\;\;0.1539\;-0.0220\;-0.0220\\ \;-0.0220\;-0.0220\;-0.0220\;-0.0220\;-0.0220\;-0.0220\;\;\;\;\;\;0.1539\;-0.0220\\ \;-0.0220\;-0.0220\;-0.0220\;-0.0220\;-0.0220\;-0.0220\;-0.0220\;\;\;\;\;\;0.1539 \end{array}\right]I$$

Combining Table 3 and Equation (32), the magnetic field intensity values generated by each magnetic pole under the three working conditions can be obtained, as shown in Table 6.

According to the angles of each magnetic pole in the coordinate system as shown in Figure 1, it can be obtained that

(33) $$\frac{\partial D(0,0)}{\partial x}=\frac{A}{2\mu_{0}}diag[\begin{array}{llllllll} 1 & 0.707 & 0 & -0.707 & -1 & -0.707 & 0 & 0.707] \end{array}$$

(34) $$\frac{\partial D(0,0)}{\partial y}=\frac{A}{2\mu_{0}}diag[\begin{array}{llllllll} 0 & 0.707 & 1 & 0.707 & 0 & -0.707 & -1 & -0.707] \end{array}$$

Based on the magnetic field strengths corresponding to the three coil working conditions in Table 3, Simultaneous Equations (7), (33) and (34), the calculation results corresponding to the actual EMF are shown in Table 7.

Obviously, the following conclusions can be drawn through the above theoretical calculation: for an eight-pole magnetic bearing with a redundant structure, considering no coils fail, the 8th coil fails, the 6–8th coils fail and so on, the CDM can be used to achieve the reconstruction of the EMF. For the expected EMF 8N that is generated in the x direction, the error rate is approximately 0.012%, while there are no coil failures and while the 8th coil fails, and approximately 0.037% while the 6–8th coils fail. For the failure of the actuator, this conclusion reflects the rationality of fault tolerance control that is based on the CDM of the magnetic bearing with a redundant structure.

### 3.3. Simulation of Fault-Tolerant Control

**Figure 3:**
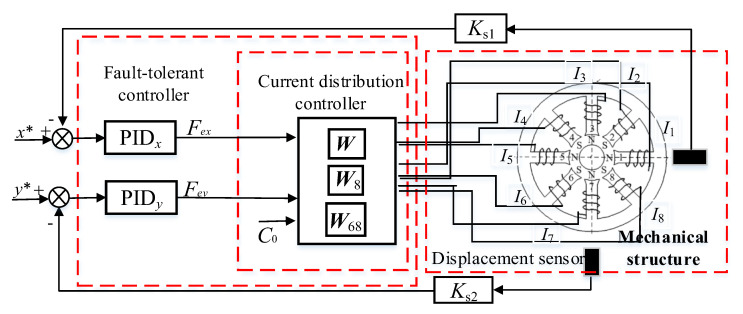
Fault−tolerant control block diagram of the magnetic bearing with redundant structure.

Support reconstruction is the theoretical basis for achieving the fault-tolerant control of the magnetic levitation rotor system while the coil fails. The control process is simulated by version 2018b of Matlab/Simulink, and the control block diagram of magnetic bearing is described in Figure 3. The position controller adopts a PID algorithm, and they are defined as PID*x* and PID*y*, respectively. The PID is

(35) $${\mathrm{PID}}_{x}(s)=k_{p}+\frac{k_{i}}{s}+k_{d}s$$

The parameters of PID*_x_* and PID*_y_* are $k_{px}=k_{py}=5$, $k_{ix}=k_{iy}=0.1$, $k_{dx}=k_{dy}=0.05$. The gain of sensors is 5000 V/m, and *C* = 4. Set the disturbing force of the rotor in the *x* and *y* directions $f_{x}=8\sin\left(100\pi t\right)$ *N* and $f_{y}=5\sin\left(100\pi t+45{}^{\circ}\right)$ *N*.

**Figure 4:**
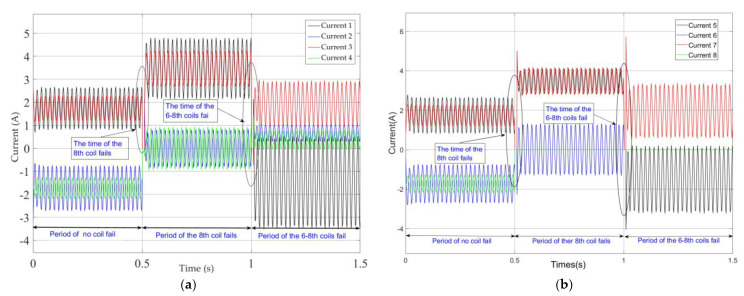
Currents of coils while the rotor speed is 2400rpm under improved fault−tolerant control. (**a**) Current change curves in the 1–4th coils. (**b**) Current change curves in the 5–8th coils.

**Figure 5:**
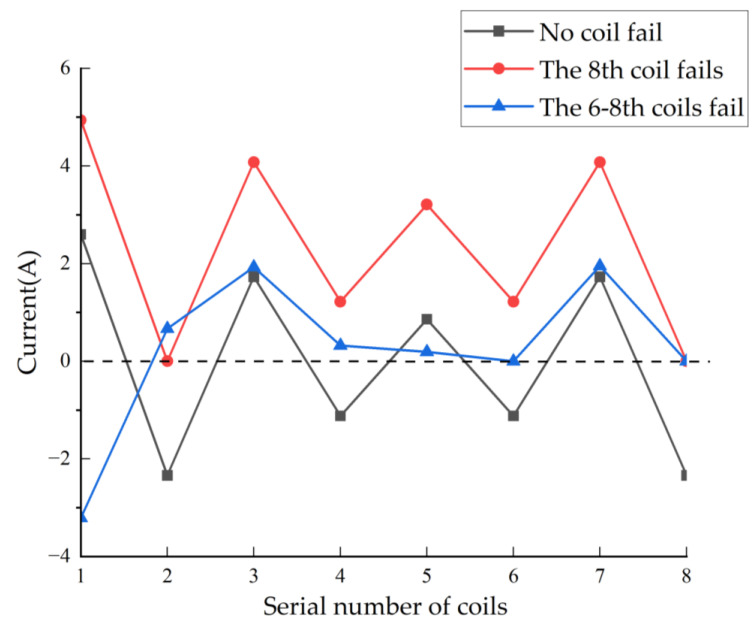
Amplitude of currents of each coil under three operating conditions.

**Figure 6:**
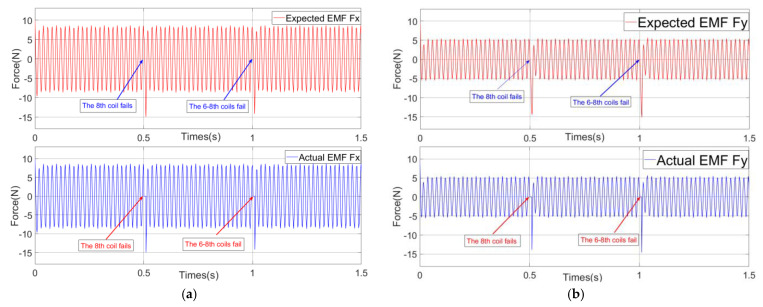
Curves of expected EMF and actual EMF: (**a**) EMF in the *x* direction and (**b**) EMF in the *y* direction.

**Figure 7:**
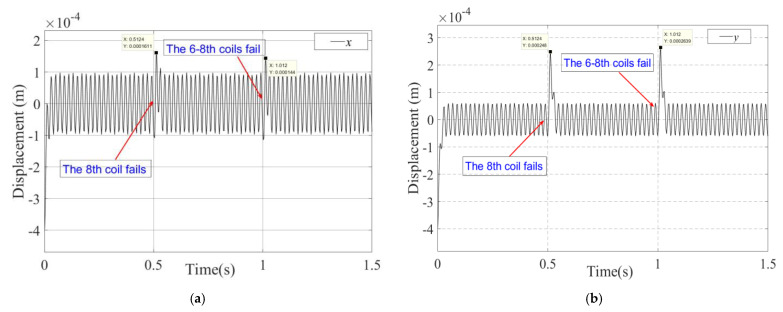
The rotor vibration displacement curves under fault-tolerant control (the state includes no coil fail, the 8th coil fails and the 6–8th coils fail): (**a**) Displacement of the rotor in the *x* direction. (**b**) Displacement of the rotor in the *y* direction.

This paper will verify the essence of the support reconstruction theory from three aspects: the changes in the current of each coil, EMF, and rotor displacement under fault-tolerant control. During the simulation process, at the initial time, there is no coil failure, the 8th coil fails at 0.5 s and the 6–8th coils fail at 1.0 s. Among these, the variation curves and amplitude of current in each coil are shown in Figure 4 and Figure 5, and the expected and actual force variation curves in the *x* and *y* directions are shown in Figure 6. Meanwhile, the vibration displacement curves of the rotor are shown in Figure 7.

As shown in Figure 4 and Figure 5, the current change characteristics in the coil not only reflect the fault conditions of the magnetic pole coils but also reveal the change patterns of the residual magnetic pole coil current characteristics after partial magnetic pole coil failures occur. By analyzing the current characteristics of the coil, we can gain a clearer understanding of the fault-tolerant control theory for magnetic bearings with redundant structure. Figure 4 shows the variation in the current in the eight magnetic pole coils of the magnetic bearing during the sinusoidal disturbance simulation. At 0.5 s, the current of the 8th coil becomes 0 A, indicating a fault in the coil. At this moment, since the fault-tolerant controller switches to the current distribution controller corresponding to the fault of the 8th coil fails, the current values of the remaining magnetic pole coils have undergone a sudden change, forming a matching support characteristic. Subsequently, the effect of maintaining the rotor in a suspended state under the fault of the magnetic pole coil was achieved. Furthermore, at time 1 s, the 6th and 8th coils malfunction, and the corresponding current in the magnetic pole coils changes to 0 A. Figure 5 shows the situation where the current values of the 6th and the 8th coils are 0 A.

As shown in Figure 6, throughout the entire simulation process, the actual EMF generated by the magnetic bearings in the *x* and *y* directions always follow the expected EMF. Especially at the moment while the coil fails, the expected EMFs are still effectively generated, which effectively verifies the EMF reconstruction theory of the magnetic bearing with redundant structure.

As shown in Figure 7, while faced with different coil failures, the fault-tolerant controller enables the bearing to achieve support reconstruction, which is mainly reflected in the following aspects: At 0.5 s, a fail occurred in the 8th coil (current value became 0), causing the bearing to undergo an instantaneous support reconstruction process. The rotor experienced a stable-unstable-stable state transition, with maximum displacements of 1.6 × 10^−4^ m and 2.8 × 10^−4^ m in the *x* and *y* directions, respectively. At time 1 s, a failure occurred simultaneously in the 6th and 8th coils, and the rotor suspension state was reconfigured again, with maximum displacements of 1.4 × 10^−4^ m and 2.6 × 10^−4^ m in the *x* and *y* directions, respectively. The above simulation results validate the effectiveness of the magnetic bearing with redundant structure support reconfiguration theory.

Based on the above results and analysis, the control system adopts the corresponding current distribution controller, which again causes the current in the residual magnetic pole coils to change, thereby reconstructing the support characteristics of the magnetic levitation bearing and forming a matching rotor suspension condition to continue maintaining the rotor’s suspension state.

## 4. Conclusions

This paper first conducts a theoretical analysis of bias current linearization using a single-degree-of-freedom magnetic levitation body as an example, clarifying the concept and function of the CDM. Then, this paper constructs a nonlinear EMF model for an eight-pole magnetic bearing with a redundant structure and uses the bias current linearization theory to express the EMF in a linearized form. Furthermore, taking the examples of no coils fail, the 8th coil fails, and the 6th to 8th coils fail, the corresponding current matrix is solved using the maximum principle function of EMF function and the numerical calculation method. At the same time, based on the current distribution matrices under the three working conditions, the corresponding magnetic flux densities were calculated, respectively, in this paper, and it was proven that the reconstruction of EMF could be achieved under the three supporting structures. Finally, a fault-tolerant control system was constructed based on the CDM and position control law. The simulation results revealed the mechanism of support reconstruction from three aspects: rotor displacement, magnitude, and direction of current in each coil. The work and results of this paper can provide valuable reference for the working mechanism and related control research of redundant structure magnetic suspension bearings.

This study has achieved at most two coil-supporting reconstructions under fault conditions, but it still has limitations. This study did not take into account the influence of thermal effect compensation and system delay on the support reconstruction process. When the rotor operates at high speed, the temperature rise in the coil will cause a change in its resistance, resulting in an increase in system power loss and even affecting system stability. At the moment the fault occurs, the rotor still has a certain transient displacement. We will continue to optimize the control algorithm for this problem to reduce the dynamic delay of the support reconstruction process. In the future, in order to further improve the real-time performance and stability of support reconstruction, we consider introducing a sliding mode observer (SMO) to predict unmodeled disturbances. For the nonlinear problem of EMF, we hope to solve it through H_∞_ control or neural network control algorithms. In terms of thermal effect compensation, we will establish a coupling model of coil temperature rise and EMF to achieve compensation for the loss of thermal effect.

## Figures and Tables

**Table 1:** The parameters of magnetic bearing.

Parameters	Physical Meaning	Parameters	Physical Meaning
$R_{j}$	Reluctance of the air gap	$N_{j}$	Turns of the coil
$g_{j}$	Length of the air gap	$I_{j}$	Current of the *j*-th coil
$A_{j}$	Pole area	*μ*_0_	Permeability of the vacuum
$\phi_{j}$	Magnetic flux		

**Table 2:** Structural parameters of magnetic bearing.

Structure Parameter	Value	Unit
Pole area, *A*_0_	5.4 × 10^−5^	m^2^
Turns per coil, *N*	56	/
Pole initial gap, *g*_0_	4 × 10^−4^	m
Pole angle, *θ_j_*	(*j* − 1)π/4	rad
Saturation magnetic-flux density, *B_sat_*	1.2	T
Rotor weight, *m*	0.8	kg

**Table 3:** The corresponding current value of each coil in three working conditions, while the EMFs are *F_x_* = 8 N, *F_y_* = 0 N.

	*I*_1_ (*A*)	*I*_2_ (*A*)	*I*_3_ (*A*)	*I*_4_ (*A*)	*I*_5_ (*A*)	*I*_6_ (*A*)	*I*_7_ (*A*)	*I*_8_ (*A*)
No coils fail	2.60	−2.34	1.73	−1.12	0.86	−1.12	1.73	−2.34
The 8th coil fails	4.94	0	4.08	1.22	3.21	1.22	4.08	0
The 6–8th coils fail	−3.21	0.67	1.93	0.32	0.19	0	1.95	0

**Table 4:** RMS current comparison.

Working Conditions	*I_RMS_* (*A*)	Differences
No coils fail	11.50	
The 8th coil fails	18.75	63%
The 6–8th coils fail	8.27	−28%

**Table 5:** Power loss comparison.

Working Conditions	*P* (*W*)	Differences
No coils fail	66.13	
The 8th coil fails	175.78	166%
The 6–8th coils fail	34.20	−48%

**Table 6:** The corresponding magnetic field strength values of each coil in three working conditions when the EMF is *F_x_* = 8 N, *F_y_* = 0 N.

	*B*_1_ (*T*)	*B*_2_ (*T*)	*B*_3_ (*T*)	*B*_4_ (*T*)	*B*_5_ (*T*)	*B*_6_ (*T*)	*B*_7_ (*T*)	*B*_8_ (*T*)
No coils fail	0.45	−0.41	0.30	−0.19	0.15	−0.19	0.30	−0.41
The 8th coil fails	0.45	−0.41	0.30	−0.19	0.15	−0.19	0.30	−0.41
The 6–8th coils fail	−0.60	0.07	0.29	0.02	0.01	−0.04	0.30	−0.04

**Table 7:** The EMF of the magnetic bearing under three working conditions.

	Expected EMF	No Coils Fail	The 8th Coil Fails	The 6–8th Coils Fail
$F_{x}$ (*N*)	8	7.999	7.999	7.997
$F_{y}$ (*N*)	0	0	0	0

## Data Availability

The original contributions presented in this study are included in the article.

## Abbreviations

The following abbreviations are used in this manuscript:

EMF	Electromagnetic Force
CDM	Current Distribution Matrix
*F_x_*, *F_y_*	Electromagnetic force in *x*/*y* Direction
*C*_0_	Bias Current Coefficient
*i_x_*, *i_y_*	Control Logic Current in *x*/*y* Direction
*B_j_*	Magnetic Flux Density at the *j*-th Pole
*B_sat_*	Saturation Magnetic Flux Density
*M_x_*, *M_y_*	The Relationship Matrix between Current and EMF
*θ**_j_*	Angular position of the *j*-th Pole
PID	Proportional–Integral–Derivative control
